# Effect of Glutathione Depletion on Ifosfamide Nephrotoxicity in Rats

**Published:** 2008-09

**Authors:** Sudha Garimella-Krovi, James E. Springate

**Affiliations:** *Department of Pediatrics, School of Medicine and Biomedical Sciences, State University of New York at Buffalo, USA*

**Keywords:** ifosfamide, kidney, glutathione, buthionine sulfoximine, toxic nephropathy

## Abstract

Kidney injury is an important side effect of the chemotherapeutic agent ifosfamide in humans. Previous studies have shown that treatment with ifosfamide reduces kidney glutathione and that the toxicity of ifosfamide is enhanced in glutathione-depleted renal tubule cells *in vitro*. In this study, we examined the effect of glutathione depletion on ifosfamide nephrotoxicity *in vivo* using rats treated with the glutathione-depleting agent buthionine sulfoximine. Animals received 80 mg/kg ifosfamide intraperitoneally daily for three days with or without buthionine sulfoximine in drinking water. Buthionine sulfoximine produced a significant fall in renal glutathione content but did not affect kidney function. Ifosfamide-treated rats developed low-grade glucosuria, phosphaturia and proteinuria that worsened with concomitant buthionine sulfoximine therapy. These findings indicate that glutathione depletion exacerbates ifosfamide nephrotoxicity in rats and suggest that pharmacological methods for replenishing intracellular glutathione may be effective in ameliorating ifosfamide-induced renal injury.

## INTRODUCTION

Ifosfamide (IFO) is a nitrogen mustard alkylating agent commonly used in chemotherapy regimens for solid tumors of bone, lung and soft tissue in children as well as adults. Its use was initially limited by the development of severe hemorrhagic cystitis. This was ameliorated by the concurrent administration of mesna (sodium 2- mercaptoethanesulfonate), a synthetic thiol that combines with reactive IFO metabolites to form stable nontoxic thioether compounds. However, despite receiving mesna, most IFO-treated children develop a renal tubulopathy and 5-9% develop a severe persistent De Toni-Debre-Fanconi syndrome (1, 2). This syndrome is caused by a general dysfunction of renal proximal tubule cells and is defined clinically by excessive urinary excretion of glucose, amino acids, phosphate, bicarbonate and other solutes handled by this nephron segment. Growth failure, rickets and progressive renal failure are sequelae of this disorder.

**Figure 1:**
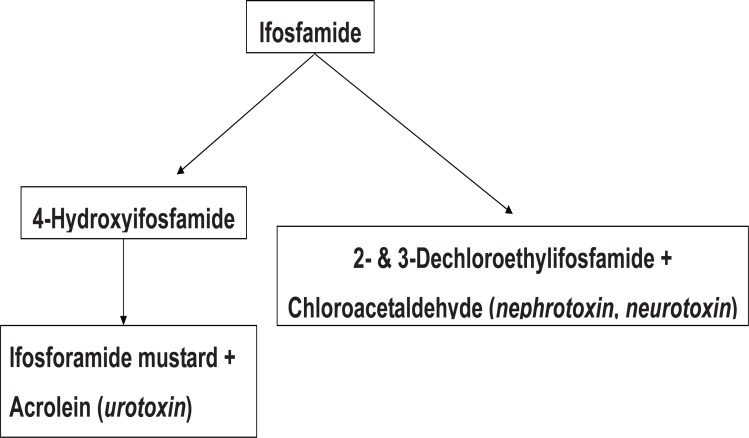
Metabolism of ifosfamide.

Interestingly, the antineoplastic medication cyclophosphamide, a structural isomer of IFO, can cause hemorrhagic cystitis but is not nephrotoxic. Both cyclophosphamide and IFO are prodrugs that must be activated by the cytochrome P450 systems to 4-hydroxy metabolites before they can exert their therapeutic or toxic effects (3). Subsequent metabolism leads to the formation of alkylating mustard derivatives and acrolein, the putative cause of hermorrhagic cystitis. Unlike cyclophosphamide, IFO also undergoes considerable oxidation of chloroethyl side chains, with liberation of chloroacetaldehyde (CAA) (Figure 1). Because of this metabolic difference, substantially more CAA is produced from IFO than from cyclophosphamide. Current evidence indicates that CAA plays an important role in the nephrotoxicity seen with IFO but not cyclophosphamide (4, 5).

Previous studies have shown that treatment with IFO or CAA reduces kidney glutathione, a substance that plays a key role in protecting cells against many types of toxic injury (6-9). In addition, the toxicity of CAA is enhanced in glutathione-depleted renal tubule cells and addition of exogenous glutathione prevents CAA-induced proximal tubule cell injury *in vitro* (6, 10). We therefore examined the effect of glutathione depletion on IFO nephrotoxicity *in vivo* using rats treated with the glutathione-depleting agent buthionine sulfoximine (BSO).

## METHODS

### Experimental design

A total of 26 12-week-old female LEW rats were used for this study. Animals were maintained on standard rat chow and tap water. Rats were acclimatized to laboratory conditions for seven days before initiating the study. One group of animals (n=7) received intraperitoneal (IP) IFO at a dose of 80 mg/kg daily for three consecutive days. A second group (n=6) was treated with BSO (6.6 g per liter drinking water) for four days. A third group (n=7) was treated with IFO and BSO as described above, with BSO being added to drinking water one day before IFO therapy commenced. Control animals (n=6) received intraperitoneal injections of sterile water. Studies of kidney function were obtained one day after treatment courses were completed.

Urine was collected from animals kept in metabolism cages for 16 hours with free access to water but deprived of food. Urinary sodium, potassium, glucose, inorganic phosphorous and creatinine were determined from these samples. Blood samples were obtained from the tail vein at the end of collection periods. Hematocrit and plasma sodium, potassium and creatinine were measured in the samples. Rats were then sacrificed; their kidneys were perfused bloodless with iced phosphate-buffered saline, removed, blotted dry, weighed and processed for total glutathione content.

### Analytical methods

Plasma and urine sodium and potassium concentrations were measured by flame photometry. Creatinine in urine and plasma was determined after absorption and elution from Lloyd’s reagent (11). Urine protein concentrations were measured according to Bradford, with bovine albumin as a standard (12). Commercial kits were used to measure glucose, inorganic phosphorus and total glutathione.

### Statistical analysis

Results are given as means ± SEM. Differences between groups were analyzed using analysis of variance with the Scheffe post hoc test. *P*<0.05 was considered a significant difference.

## RESULTS

**Figure 2:**
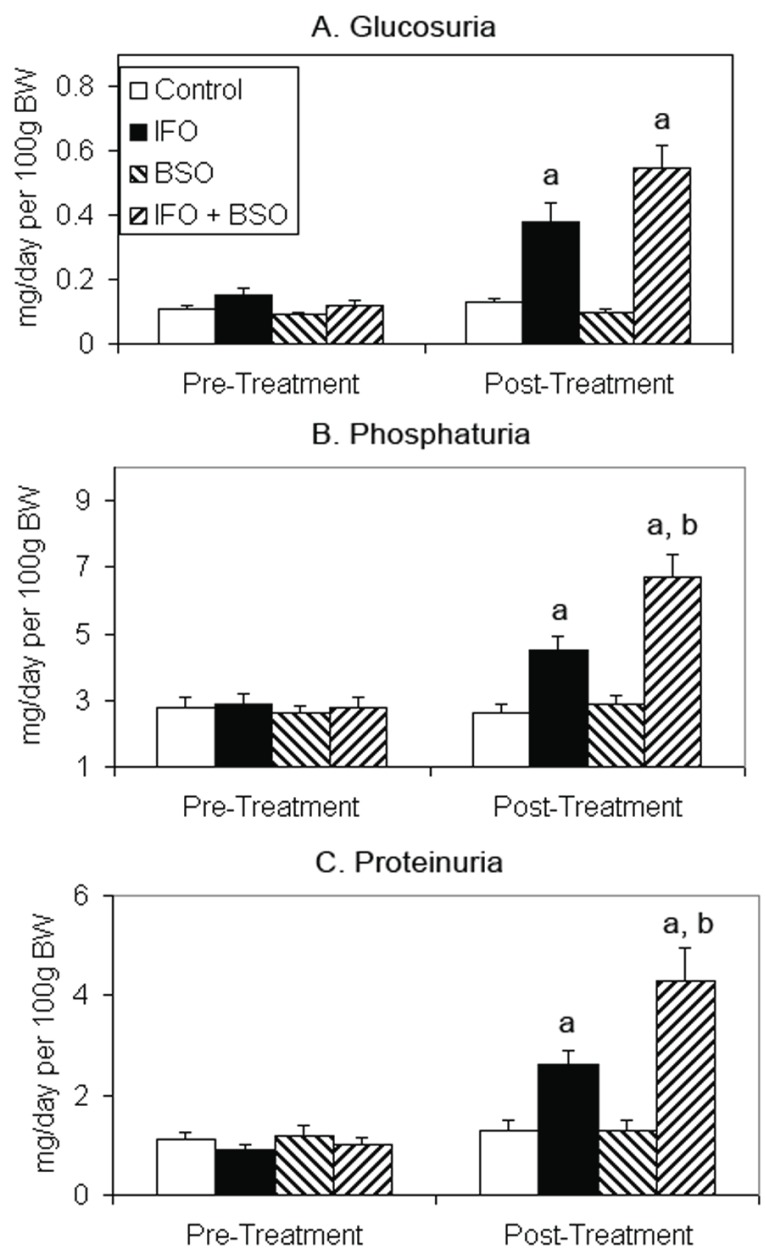
Effect of ifosfamide and buthionine sulfoximine on urinary excretion rates of glucose (A), phosphate (B) and protein (C). Results are mean ± SEM. a, *P*<0.05 vs. corresponding pre-treatment result; b, *P*<0.05 vs. post-treatment ifosfamide group.

**Figure 3:**
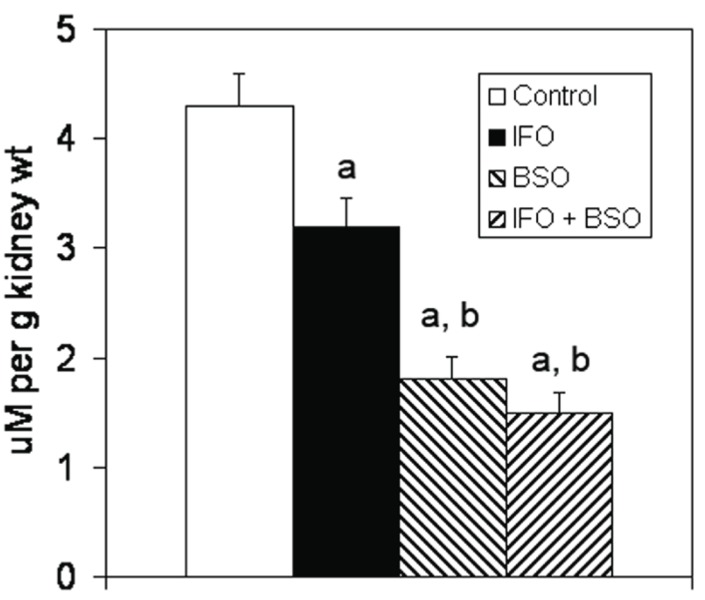
Effect of ifosfamide and buthionine sulfoximine on kidney glutathione concentrations. Results are mean ± SEM. a, *P*<0.05 vs. control group; b, *P*<0.05 vs. ifosfamide-treated group.

There were no significant differences in plasma concentrations of sodium, potassium, glucose or creatinine among groups (data not shown). Body weight, urine flow rate, creatinine clearance, and urinary sodium and potassium excretion were not affected by any treatment course (Table 1). Hematocrits of all groups of rats receiving ifosfamide were significantly lower than the control group (Table 1). Urinary glucose excretion increased significantly in the two groups of animals that received IFO but was not affected by BSO therapy (Figure 2A). Urinary phosphorus excretion was significantly greater in IFO-treated rats and increased further when IFO was combined with BSO (Figure 2B). Similar findings were noted in urinary protein excretion (Figure 2C). IFO treatment caused a mean 22% reduction in kidney glutathione concentration while BSO produced a mean 56% decline in renal glutathione (Figure 3). Rats receiving BSO alone had kidney glutathione concentrations that were not significantly different from those receiving both IFO and BSO.

**Table 1:** Effect of ifosfamide and buthionine sulfoximine on kidney function in rats

Group (n)	Body weight (kg)	Hematocrit (%)	Urine flow rate (ul/min)	Creatinine clearance (ml/min)	Urinary sodium excretion (mEq/day)	Urinary potassium excretion (mEq/day)

Control (6)	212 ± 7	49 ± 1	0.67 ± 0.05	0.72 ± 0.07	0.29 ± 0.05	0.32 ± 0.05
IFO (8)	205 ± 6	46 ± 1^a^	0.74 ± 0.06	0.61 ± 0.06	0.32 ± 0.06	0.38 ± 0.06
BSO (6)	210 ± 6	50 ± 1	0.59 ± 0.05	0.71 ± 0.05	0.27 ± 0.09	0.37 ± 0.03
BSO + IFO (8)	202 ± 6	45 ± 1^a^	0.71 ± 0.04	0.66 ± 0.10	0.39 ± 0.12	0.40 ± 0.03

Results are mean ± SEM;

a P<0.05 vs. control group.

## DISCUSSION

The present study demonstrates that rats treated with IFO develop kidney glutathione depletion and abnormalities in renal function resembling human Fanconi syndrome. These findings are generally consistent with previous studies. For example, Nissim and Weinberg (7) administered IFO 50 mg/kg IP daily for five days to male Sprague-Dawley rats and noted significant glutathione depletion, reduction in creatinine clearance and hyperphosphaturia. Both Badary (8) and Sehrli (9) treated Wistar rats with 50 mg/kg IFO IP daily for five days and found kidney glutathione depletion together with reduced creatinine clearance, hyperphosphaturia, glucosuria and proteinuria. Finally, Appenroth *et al*. (13) gave IFO 80 mg/kg IP daily for three days to adult female Wistar rats with subsequent development of proteinuria and hyperphosphaturia.

*In vitro* studies have suggested that depletion of glutathione is a key determinant in IFO-induced nephrotoxicity (6, 14, 15). The present study supports this impression *in vivo* by demonstrating worsening of renal tubular function when IFO is administered to glutathione depleted rats. Glutathione depletion with BSO alone had no effect on renal function as previously noted by others (16).

It is of interest in this regard that mesna prevents IFO nephrotoxicity *in vitro* yet this uroprotectant medication does not prevent renal injury in clinical practice. After intravenous infusion, mesna is rapidly oxidized to the inactive disulfide, dimesna (17). The liver recycles dimesna to mesna so that both forms are present in the systemic circulation (18). Both mesna and dimesna are cleared from the circulation by glomerular filtration. Dimesna is reabsorbed in the proximal nephron, reduced to mesna and secreted back into the urine where it combines with reactive IFO metabolites to form stable nontoxic thioether compounds thus preventing hemorrhagic cystitis. Conversion of dimesna to mesna by proximal tubule cells consumes glutathione (17, 19). The paradoxical possibility therefore exists that mesna-induced renal glutathione depletion contributes to IFO nephrotoxicity.

There is only limited information about the effect of mesna on kidney glutathione. Springate (20) found that mesna significantly reduced intrarenal total glutathione in rats while Kabasakal *et al*. (21) found no significant effect on renal total glutathione concentration. However, the relative proportions of reduced (biologically active) glutathione and oxidized glutathione that make up total glutathione were not measured in either study. The effect of typical clinical mesna administration regimens on renal glutathione levels remain to be determined.

In conclusion, the present study demonstrates that glutathione depletion exacerbates IFO nephrotoxicity in rats. Our findings support the hypothesis that glutathione protects against IFO toxicity and that pharmacological methods for replenishing intracellular glutathione may be effective in modulating IF-induced renal injury (14, 15).
